# Subgingival Microbiota Shifts Following Diode Laser-Activated Indocyanine Green Treatment in Periodontitis: A Pilot 16S rDNA Study

**DOI:** 10.3390/microorganisms14061347

**Published:** 2026-06-16

**Authors:** Dimitra Diakoumopoulou, Aleksandra Slavko, Konstantinos Papadimitriou, Ioannis K. Karoussis, Chrysoula Nikolaou, Stylianos Chatzipanagiotou, Anastasios Ioannidis

**Affiliations:** 1 Department of Clinical Microbiology, Athens Medical School, Aeginition Hospital, National and Kapodistrian University of Athens, 11528 Athens, Greece; ddiakoumopoulou@hotmail.com (D.D.); chrysoula.nikolaou@gmail.com (C.N.); schatzipa@gmail.com (S.C.); 2 Department of Food Science and Technology, School of Agriculture and Food, University of the Peloponnese, 24100 Kalamata, Greece; a.slavko@go.uop.gr; 3 Laboratory of Food Quality Control and Hygiene, Department of Food Science and Human Nutrition, Agricultural University of Athens, Iera Odos 75, 11855 Athens, Greece; kpapadimitriou@aua.gr; 4 Department of Periodontology, School of Dental Medicine, National and Kapodistrian University of Athens, 11527 Athens, Greece; karoussisperio@gmail.com; 5 Laboratory of Clinical Microbiology, School of Medicine, Attikon University Hospital, National and Kapodistrian University of Athens, Rimini 1, 12462 Chaidari, Greece

**Keywords:** periodontal disease, EmunDo, photosensitiser, 16S rDNA sequencing, subgingival microbiome, periodontal pathogens

## Abstract

Periodontal disease is driven by a dysbiotic subgingival microbiota enriched in anaerobic pathogens, and novel antimicrobial strategies are needed to complement conventional therapy. This pilot study assessed changes in the subgingival microbiota following diode laser-activated indocyanine green-based treatment (EmunDo) using 16S rDNA amplicon sequencing of paired samples collected before and after therapy. Microbiome analysis revealed compositional shifts across all taxonomic levels, with reductions in disease-associated genera including *Porphyromonas*, *Treponema*, *Fretibacterium*, and *Prevotella*, and relative increases in taxa more commonly associated with periodontal health, such as *Streptococcus*, *Actinomyces*, and *Haemophilus*. Functional prediction further suggested treatment-associated variation in metabolic categories. Overall microbial richness was preserved between groups. These findings suggest that EmunDo treatment was associated with a restructuring of the subgingival microbiota toward a less dysbiotic profile, warranting further investigation in larger controlled studies using higher-resolution approaches such as shotgun metagenomics.

## 1. Introduction

Periodontitis is a chronic inflammatory disease that affects the supporting tissues of the teeth and is among the most prevalent oral diseases worldwide. It is characterized by periodontal pocket formation, loss of attachment, and progressive alveolar bone destruction, which may ultimately result in tooth loss if untreated [1]. Epidemiological studies indicate that periodontitis affects a large proportion of the adult population globally and represents a major public health burden due to its high prevalence and its association with systemic conditions such as diabetes, cardiovascular diseases and premature birth [2].

The etiology of periodontitis is strongly associated with the accumulation of complex microbial biofilms in the subgingival environment [3,4]. The oral cavity hosts a highly diverse microbial community, and under healthy conditions, a dynamic equilibrium exists between the resident microbiota and host immune responses. However, disruption of this balance leads to microbial dysbiosis, which triggers inflammatory responses that contribute to periodontal tissue destruction [1,4]. Several bacterial taxa have been associated with periodontal disease progression, including *Porphyromonas gingivalis*, *Tannerella forsythia* and *Treponema denticola*, which constitute the red complex strongly linked to advanced periodontal destruction [5,6]. In addition, orange-complex bacteria, such as *Prevotella intermedia* and *Fusobacterium nucleatum*, are important bridging organisms that facilitate pathogenic biofilm maturation [1,5,7]. In contrast, yellow-complex microorganisms, including *Streptococcus* spp., and green-complex members, such as *Capnocytophaga* spp., are more frequently associated with periodontal health [5,6]. However, shifts in the relative abundance of these commensal species, together with increased colonization by pathogenic complexes, can promote dysbiotic microbial communities and periodontal inflammation. This ecological shift involves loss of health-associated bacteria and expansion of opportunistic anaerobic pathogens, leading to disruption of microbial homeostasis and activation of host inflammatory responses [8]. Periodontal disease progression also involves microbial succession, with health-associated taxa decreasing while periodontitis-associated species become dominant in the subgingival biofilm [5].

Recent advances in microbiome research have highlighted the complex ecological nature of periodontal disease. Periodontitis is no longer considered a simple infection caused by a limited number of pathogens but rather a polymicrobial dysbiotic condition involving shifts in microbial community structure and bacterial interactions [1]. High-throughput sequencing studies have demonstrated that periodontal pockets harbor communities with increased diversity and distinct composition compared with healthy sites, emphasizing the importance of microbial dynamics in disease progression [2]. Next-generation sequencing (NGS) has further shown that periodontal pockets include emerging pathogens not originally described within the classical Socransky complexes [6,9,10]. Among these, *Filifactor alocis* and *Fretibacterium* spp. have been associated with periodontal disease and appear to be highly prevalent in deep periodontal pockets [11,12]. These microorganisms are considered important members of dysbiotic periodontal communities and may contribute to disease progression through interactions with established periodontal pathogens and modulation of host immune responses.

Conventional nonsurgical periodontal therapy is based primarily on mechanical supra- and subgingival instrumentation for biofilm and calculus removal, which represents a central component of the stepwise treatment approach recommended for stage I–III periodontitis [13]. Mechanical therapy can significantly reduce bacterial load and improve clinical periodontal parameters. However, the anatomical complexity of periodontal pockets and root surfaces makes complete elimination of pathogenic microorganisms difficult [14]. Although periodontal treatment alters the composition of the subgingival microbiome and reduces dysbiosis, microbial communities may gradually recover and re-establish biofilms within days or weeks after treatment [2]. Adjunctive therapeutic approaches, including systemic antibiotics and locally delivered antimicrobials, have therefore been proposed to enhance conventional periodontal therapy. Such treatments can further reduce periodontal pathogens and shift the microbiome toward a healthier state [15].

In recent years, light-based antimicrobial therapies have attracted increasing interest as adjunctive approaches in periodontal treatment. Among these, photodynamic therapy (PDT) has emerged as a promising non-invasive method [16,17,18]. PDT involves the interaction between a photosensitizer (PS), light of a specific wavelength, and molecular oxygen, leading to the production of reactive oxygen species (ROS) that damage bacterial cell membranes, proteins, and nucleic acids [17,19]. Among the PS used, indocyanine green (ICG)-based systems, including EmunDo treatment, have attracted attention because of their strong near-infrared absorption and ability to enhance bacterial inactivation mainly through photothermal effects, with possible additional photodynamic contributions upon laser activation [16,20]. Although EmunDo has been used in veterinary applications, previous studies have also investigated EmunDo and related ICG-based 810 nm diode laser protocols as adjunctive approaches for reducing periodontal bacterial burden [19,21,22,23,24,25].

Despite these promising findings, most studies evaluating light-based antimicrobial therapies in periodontitis have focused on the reduction in specific target pathogens, while culture-independent characterization of treatment-associated community-level changes remains limited [21,26,27]. High-throughput 16S rDNA amplicon sequencing allows comprehensive profiling of oral microbial communities and detection of treatment-associated bacterial shifts that may be missed by culture-based or targeted molecular approaches [28]. Therefore, the aim of the present study was to assess treatment-associated changes in subgingival bacterial community composition following laser-activated EmunDo treatment using 16S rDNA amplicon sequencing of samples collected before and after treatment.

## 2. Materials and Methods

### 2.1. Samples

Subgingival plaque samples for microbiome analysis using 16S rDNA sequencing were collected from four patients diagnosed with periodontitis before (BF) and after (AF) the treatment. All the patients, 2 males and 2 females, systemically healthy, non-smokers, were diagnosed with periodontitis Stage III Grade 2. The age of the patients ranged from 47 to 72 years. Before laser treatment with ICG, all the patients received conventional non-surgical periodontal treatment, including scaling and root planing, for the treatment and prevention of periodontal disease progression. Treatment was performed using the EmunDo system (A.R.C. Laser GmbH, Nürnberg, Germany), which combines ICG as a near-infrared PS with activation by a diode laser (Fox, A.R.C. Laser GmbH, Nürnberg, Germany). The ICG solution at a concentration of 1 mg/mL was applied into the periodontal pocket and left in situ for approximately 60 s before laser irradiation to allow interaction with the subgingival biofilm. Following application of the PS to the periodontal pocket, laser irradiation was delivered via a bulb-shaped optical fiber tip suitable for periodontal pocket application. The optical fiber diameter was approximately 300–400 μm. Laser irradiation was performed in continuous-wave mode at 810 nm, with a power output of 500 mW for 30 s per treatment site. The estimated spot area was 0.5 cm^2^, corresponding to an energy density of 30 J/cm^2^ per treatment site, calculated as follows: (0.5 W × 30 s)/0.5 cm^2^ = 30 J/cm^2^. The laser output was based on the device settings. In each patient, up to four periodontal sites were treated and sampled. The sterile paper points were inserted into the deepest periodontal pockets, with pocket probing depth (PPD) ranging between 6 mm and 8 mm. Sampling was performed using sterile paper points inserted into periodontal pockets and immediately transferred into sterile tubes. In each patient, up to four periodontal sites were treated and sampled. The sterile paper points were inserted into the deepest periodontal pockets, with PPD ranging between 6 mm and 8 mm. To minimize contamination of subgingival samples with supragingival plaque, supragingival plaque was carefully removed from the tooth surface using sterile paper points before sampling, the area was isolated and air-dried, and care was taken to avoid contact with adjacent tissues during sample collection. For each patient, paper points collected from the selected periodontal sites were pooled into a single sample for microbiome analysis at each time point, generating one BF and one AF pooled sample per patient. After collection, the samples were cooled at −20 °C and subsequently stored at −80 °C until further processing. The AF reevaluation sampling was performed 8 weeks after treatment, at the same sites as BF.

### 2.2. Sequencing and Bioinformatics Analysis

Metagenomic DNA was extracted from the BF and AF groups of samples. DNA extraction, library preparation, 16S rDNA gene sequencing and initial quality control were performed by BGI Genomics (Shenzhen, China) using the DNBseq sequencing platform with paired-end reads of 300 bp. Following sequencing, read quality was assessed with FastQC and the reads were subsequently imported to CLC Genomics Workbench v25.0.3 (Qiagen, Hilden, Germany) for downstream analysis, including chimera removal and operational taxonomic unit (OTU) clustering using the default parameters. Taxonomic classification was performed by aligning the sequences against the SILVA 138.1 database, with reads assigned to bacterial taxa with a 97% sequence similarity threshold. Alpha diversity was evaluated using rarefaction curves and by calculating the observed number of genera following rarefaction based on the total number of genera, while beta diversity was assessed using principal coordinate analysis (PCoA) based on Bray–Curtis dissimilarity. Heatmaps were generated at the genus level to visualize differences in the relative abundance of bacterial taxa across samples. Core microbiome analysis using a minimum relative abundance threshold of 0.01% and a sample prevalence threshold of 20%, and the linear discriminant analysis effect size (LEfSe) with a significance threshold of *p* < 0.05 and an LDA score cutoff of 2.0 were conducted at the genus level using MicrobiomeAnalyst 2.0 [29]. Predicted functional profiling was performed using PICRUSt2 v2.5.3 with default settings [30]. Predicted MetaCyc pathways were grouped into functional categories, values were ln-transformed and visualized in ClustVis v2.0 as a heatmap [31].

## 3. Results and Discussion

### 3.1. Microbiome Analysis Based on 16S rDNA Amplicon Sequencing

Clinically, all the sampled periodontal pockets showed improvement at the 8-week follow-up, with probing pocket depths reduced to ≤4 mm. This improvement was accompanied by compositional shifts in the subgingival microbiota before (BF) and after (AF) EmunDo treatment, suggesting treatment-associated restructuring of the microbial community.

**Figure 1:**
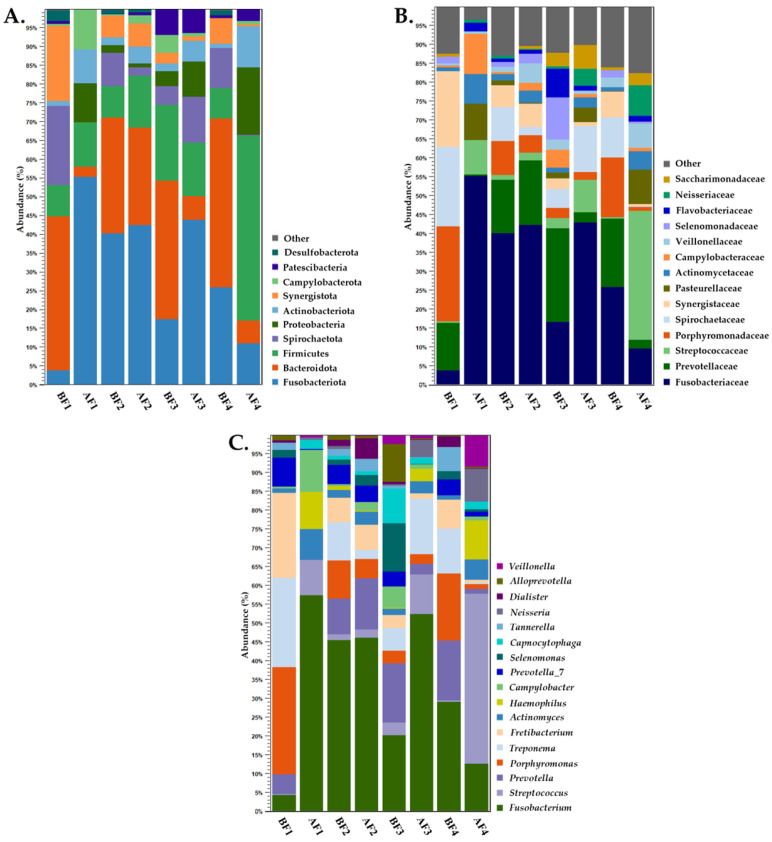
Stacked bar charts illustrating the relative abundances (%) of bacterial taxa across samples before (BF) and after (AF) EmunDo system treatment: (**A**) Phylum-level composition. (**B**) Family-level composition. (**C**) Genus-level composition of taxa with relative abundance ≥ 1%.

At the phylum level (Figure 1A), both the BF and AF samples were dominated by the same major phyla, although their relative abundances differed between individuals. In the BF samples, Bacteroidota was the most abundant phylum (31–45%), followed by Fusobacteriota (4–40%), Spirochaetota (5–21%), Firmicutes (8–20%), and Synergistota (2.8–20%). Following treatment, Fusobacteriota became the most prominent phylum in several AF samples (up to 55%), while Firmicutes and Bacteroidota remained consistently detected (up to 49% and 26%, respectively). Proteobacteria and Actinobacteriota also increased in the AF samples, reaching 18% and 11%, respectively. The higher representation of Bacteroidota in the BF samples is consistent with previous 16S-based observations of increased Bacteroidetes and reduced Actinobacteria in periodontitis-associated plaque communities [32].

Overall, these changes indicate shifts in the relative abundance of dominant taxa rather than gain or loss of specific phyla.

Comparison of community composition before and after treatment revealed shifts in several bacterial families (Figure 1B). Prior to treatment, the microbiota was dominated by dysbiosis-associated families, including Porphyromonadaceae, Prevotellaceae, Spirochaetaceae, and Synergistaceae, which reached relative abundances of up to 25%, 25%, 21%, and 20%, respectively. These families include periodontal pathogens such as *Porphyromonas*, *Treponema*, and *Prevotella*, which are frequently enriched in diseased periodontal pockets. Following treatment, these families decreased in relative abundance, consistent with antimicrobial effects on anaerobic periodontal biofilms. In contrast, families associated with early colonization and periodontal health increased, particularly Streptococcaceae, which rose from up to 2.7% in the BF samples to up to 34% in the AF samples. *Streptococcus* spp. are primary early colonizers involved in healthy dental biofilm formation and microbial homeostasis [33,34]. Actinomycetaceae also increased from up to 1.8% in the BF samples to 7.8% in the AF samples, consistent with the role of Actinomyces spp. in commensal oral biofilms and coaggregation with early colonizers [33,35]. Veillonellaceae, Neisseriaceae, Pasteurellaceae, and Flavobacteriaceae similarly increased after treatment. Veillonellaceae, Neisseriaceae, Pasteurellaceae, and Flavobacteriaceae similarly increased after treatment. These families include taxa such as Veillonella, Neisseria, and Haemophilus, which are frequently associated with health-compatible oral communities [33,35,36]. Overall, these changes may reflect partial ecological restoration toward a less dysbiotic microbial community, while Fusobacteriaceae remained relatively abundant after treatment.

Compositional changes at the genus level for taxa with relative abundances ≥ 1% are presented in Figure 1C. In the BF samples, the microbiota was dominated by periodontal disease-associated genera belonging mainly to red and orange complexes, including *Porphyromonas* (up to 25%), *Treponema* (up to 21%), *Fretibacterium* (up to 20%), *Prevotella* (up to 13%), *Tannerella* (up to 21%) and *Prevotella_7* (up to 6.8%). Following treatment, these genera decreased, with *Porphyromonas* and *Treponema* declining to up to 4.7% and 12%, respectively, *Fretibacterium* to up to 6.2%, and *Prevotella* and *Prevotella*_7 to up to 12% and 4.1%, respectively. Selenomonas and Desulfovibrio, both implicated in periodontal pathogenesis, also decreased after treatment [37,38,39]. In contrast, genera associated with health-compatible oral microbiota, including Streptococcus, Capnocytophaga, Actinomyces, Veillonella, Neisseria, and Rothia, increased in the AF samples. These genera are characteristic of early colonizing communities and are frequently detected at healthy periodontal sites [33,34,35]. This pattern is consistent with recent 16S rDNA profiling reporting enrichment of *Porphyromonas*, *Prevotella*, and *Treponema* in periodontitis, whereas *Streptococcus* and *Neisseria* were more abundant in healthy controls [32]. *Fusobacterium* remained abundant or increased in several AF samples. As a bridging organism in oral biofilm architecture, particularly *F. nucleatum*, its persistence may reflect its ecological role connecting early colonizers with late-stage anaerobic pathogens [33,40,41]. Its lower endogenous porphyrin content relative to black-pigmented anaerobes such as *Porphyromonas*, together with biofilm-associated protection and irradiation parameters, may also reduce its photosensitivity [26]. Since PDT efficacy against *Fusobacterium* depends on PS choice, light source, and irradiation dose [20], its persistence should be interpreted cautiously and in the context of its ecological function rather than as clear treatment resistance or primary pathogenicity [1].

**Figure 2:**
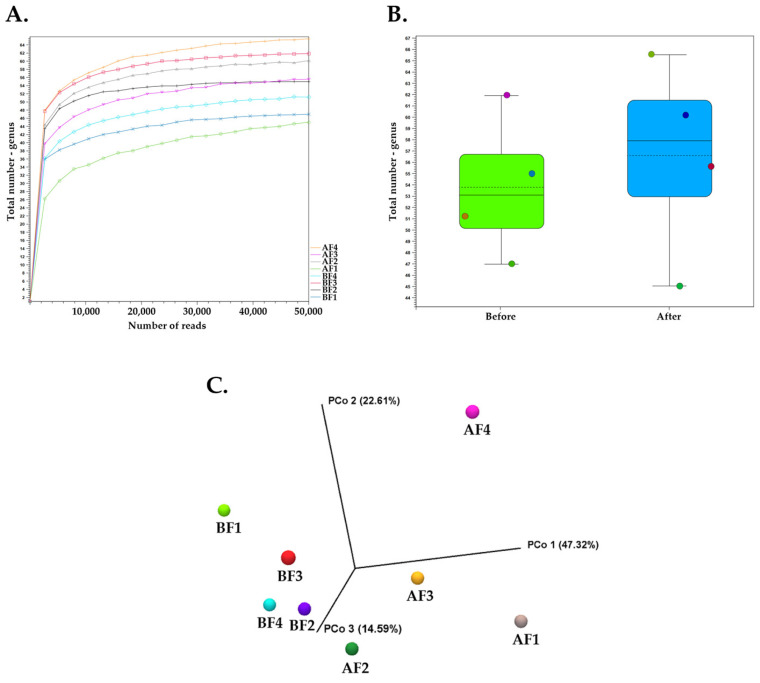
Alpha-diversity analysis of the periodontal microbiota before (BF) and after (AF) EmunDo system treatment: (**A**) Rarefaction curves showing the total number of detected genera as a function of sequencing depth (number of reads) for each sample. (**B**) Box plots showing the total number of detected genera per sample in BF (green) and AF (blue) groups. The colored spheres represent individual sample values. (**C**) Principal coordinates analysis (PCoA) of genus-level microbial community composition across samples before (BF) and after (AF) EmunDo system treatment. Each sphere represents one sample.

**Figure 3:**
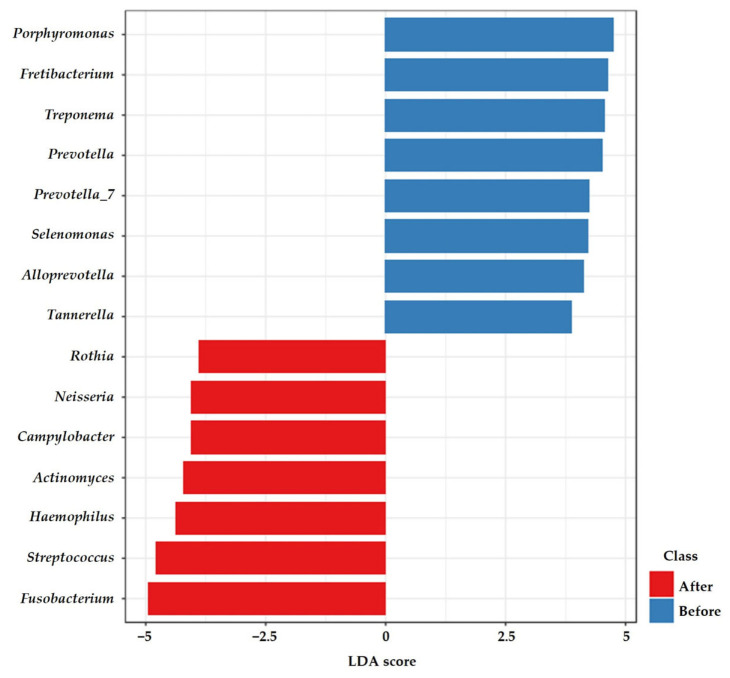
LEfSe analysis identifying bacterial genera that differed significantly in relative abundance between samples before (BF) and after (AF) EmunDo system treatment. The x-axis represents the LDA score, indicating the magnitude of the difference between groups. Genera with positive LDA scores (**right side**) were enriched in the BF samples; genera with negative LDA scores (**left side**) were enriched in the AF samples.

Rarefaction curves approached saturation for all the samples, confirming adequate sequencing depth (Figure 2A). Alpha-diversity analysis based on rarefied data (Figure 2B) revealed no statistically significant differences in microbial richness between the BF and AF samples, with the total number of detected genera ranging from 47 to 62 in BF and from 45 to 66 in the AF samples. Beta-diversity analysis (Figure 2C) showed a tendency for the BF and AF samples to cluster separately in PCoA space, further supporting treatment-driven shifts in community composition. The compositional differences between the BF and AF samples were further explored using Linear Discriminant Analysis Effect Size (LEfSe) (Figure 3). This analysis identified *Porphyromonas*, *Fretibacterium*, *Treponema*, *Prevotella*, *Prevotella*_7, *Selenomonas*, and *Alloprevotella* as the main taxa contributing to the BF profile, whereas *Fusobacterium*, *Streptococcus*, *Haemophilus*, *Actinomyces*, *Neisseria*, *Rothia*, and *Veillonella* were associated with the AF samples.

Given the limited sample size, LEfSe was interpreted as an exploratory analysis of taxa driving the observed differences rather than as definitive evidence of treatment-associated biomarkers. *Campylobacter* was also enriched in the AF samples. Members of this genus have been reported in the oral cavity and may occur under different periodontal conditions [42]. Because 16S rDNA amplicon sequencing does not provide reliable species-level resolution, the clinical significance of this signal remains unclear and requires confirmation using higher-resolution sequencing approaches.

**Figure 4:**
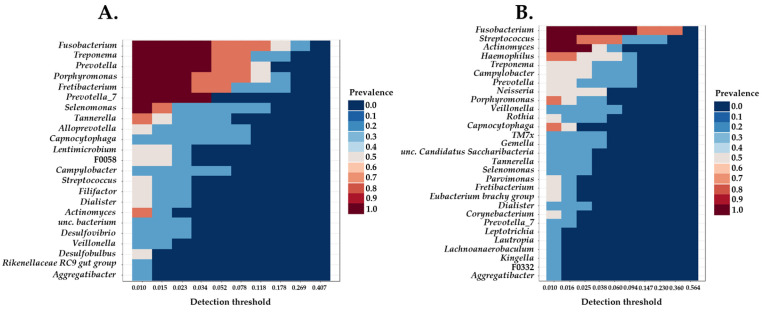
Core microbiome analysis at the genus level in samples (**A**) before and (**B**) after EmunDo system treatment. Each row represents a genus, and each column represents a detection threshold (relative abundance, %). The color scale indicates the prevalence of each genus across samples at a given detection threshold.

Core microbiome analysis (Figure 4) reinforced this picture, showing that in the BF samples the core comprised disease-associated genera including *Treponema*, *Prevotella*, *Porphyromonas*, *Fretibacterium*, *Prevotella*_7, and *Selenomonas*, whereas in the AF samples it shifted toward *Streptococcus*, *Actinomyces*, and *Haemophilus*, reflecting a clear change in the dominant and consistently present members of the community. *Fusobacterium* was detected as a core member in both groups, consistent with its role as a stable bridging organism within the periodontal microbiota.

To further explore genus-level differences before and after EmunDo treatment, a heatmap based on genus-level relative abundances was generated (Supplementary Figure S1). Hierarchical clustering showed partial grouping according to treatment status, supporting treatment-associated compositional shifts in the subgingival microbiota. Periodontitis-associated genera, including *Porphyromonas*, *Treponema*, *Prevotella*, and *Fretibacterium* showed higher relative abundance in several BF samples, whereas the AF samples showed relatively higher abundance of oral commensal genera such as *Streptococcus*, *Haemophilus*, and *Actinomyces*. Overall, the heatmap supported a compositional rearrangement after treatment, characterized by reduced abundance of selected anaerobic periodontitis-associated taxa and relative increases in taxa more commonly associated with oral health or early colonization. Together with the preservation of overall microbial richness, these findings suggest a shift toward a less dysbiotic microbiome rather than broad depletion of the microbial community.

### 3.2. Predicted Functional Profiling Based on 16S rDNA Amplicon Sequencing

**Figure 5:**
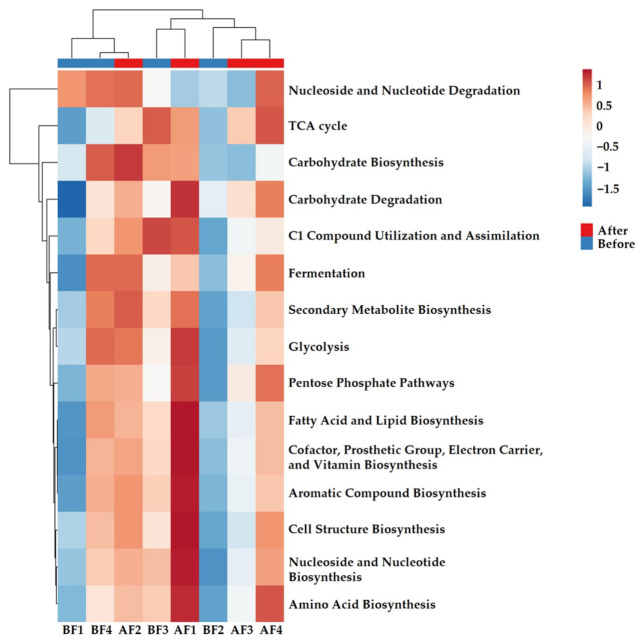
Heatmap showing the top 15 most abundant predicted functional categories for the subgingival plaque microbiota before (BF) and after (AF) treatment. The colour scale indicates relative abundance within each category, with red representing higher and blue representing lower scaled abundance.

Functional prediction revealed variation in the functional profiles between the BF and AF samples (Figure 5 and Figure S2).

The most abundant predicted categories included Nucleoside and Nucleotide Biosynthesis, Cofactor, Prosthetic Group, Electron Carrier, and Vitamin Biosynthesis, Amino Acid Biosynthesis, Fatty Acid and Lipid Biosynthesis, Cell Structure Biosynthesis, Carbohydrate Biosynthesis, Fermentation and Secondary Metabolite Biosynthesis, among others. Heatmap clustering showed partial grouping according to treatment status, with AF1 and AF4 displaying relatively higher predicted abundances of several biosynthetic and central metabolic categories compared with the BF samples. Overall, the analysis indicated sample-specific variation in inferred functional profiles, with partial treatment-associated clustering and inter-individual variability. These findings suggest that the observed taxonomic shifts were accompanied by changes in the inferred functional potential of the subgingival microbiota.

## 4. Conclusions

16S rDNA amplicon sequencing of subgingival plaque samples collected before and after laser-activated EmunDo treatment revealed treatment-associated compositional shifts in the periodontal microbiota. Before treatment, the subgingival community was dominated by genera associated with periodontal dysbiosis, including *Porphyromonas*, *Treponema*, *Fretibacterium*, *Prevotella*, and *Tannerella*. After treatment, these taxa decreased, while genera linked to early colonization and periodontal health, including *Streptococcus*, *Actinomyces*, *Haemophilus*, *Veillonella*, *Neisseria*, and *Rothia*, increased. The preservation of overall microbial richness suggests that EmunDo treatment was associated with community restructuring rather than broad depletion of the microbiota. Beta-diversity, LEfSe, and core microbiome analyses further supported a shift toward a less dysbiotic subgingival microbiome. Functional prediction indicated accompanying variation in inferred metabolic categories, although these findings require confirmation using higher-resolution approaches. Overall, these preliminary results support the potential of laser-activated EmunDo treatment as an adjunct to conventional periodontal therapy. As a pilot study, the present work is limited by the small number of participants, absence of additional control conditions, and assessment of microbiota changes at a single post-treatment time point. Larger controlled longitudinal studies, using higher-resolution approaches, such as shotgun metagenomics, are required to confirm these observations and clarify their functional significance.

## Data Availability

The 16s rDNA amplicon sequencing project data presented in this study are openly available in SRA at http://www.ncbi.nlm.nih.gov/bioproject/1458546 (accessed on 10 June 2026), Bioproject ID PRJNA1458546.

## Supplementary Materials

The following supporting information can be downloaded at https://www.mdpi.com/article/10.3390/microorganisms14061347/s1. Figure S1. Heatmap showing the relative abundance of all detected bacterial genera across samples collected before (BF) and after (AF) the EmunDo system treatment. Genera and samples were hierarchically clustered based on their relative abundance profiles. Colors represent normalized abundance values, with yellow indicating higher abundance and blue indicating lower abundance. Figure S2. Heatmap of functional categories in subgingival plaque samples before (BF) and after treatment (AF). The colour scale indicates relative abundance within each category, with red representing higher and blue representing lower scaled abundance.
