# Supplementary figures and images for: Subgingival Microbiota Shifts Following Diode Laser-Activated Indocyanine Green Treatment in Periodontitis: A Pilot 16S rDNA Study
Source: Microorganisms. 2026 Jun 16;14(6):1347. doi: 10.3390/microorganisms14061347 (PMC13304405; doi:10.3390/microorganisms14061347)

Figure S1

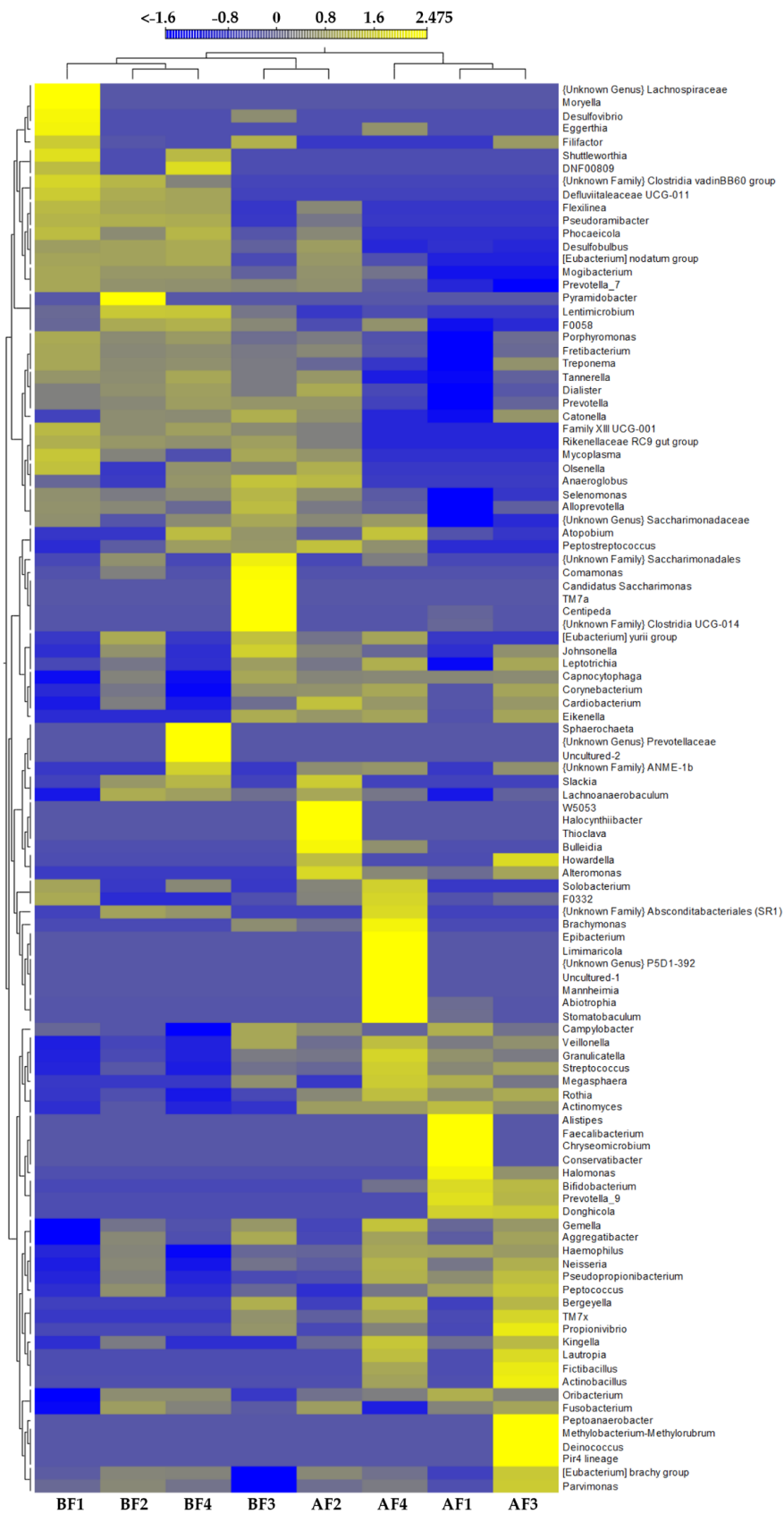

Figure S2

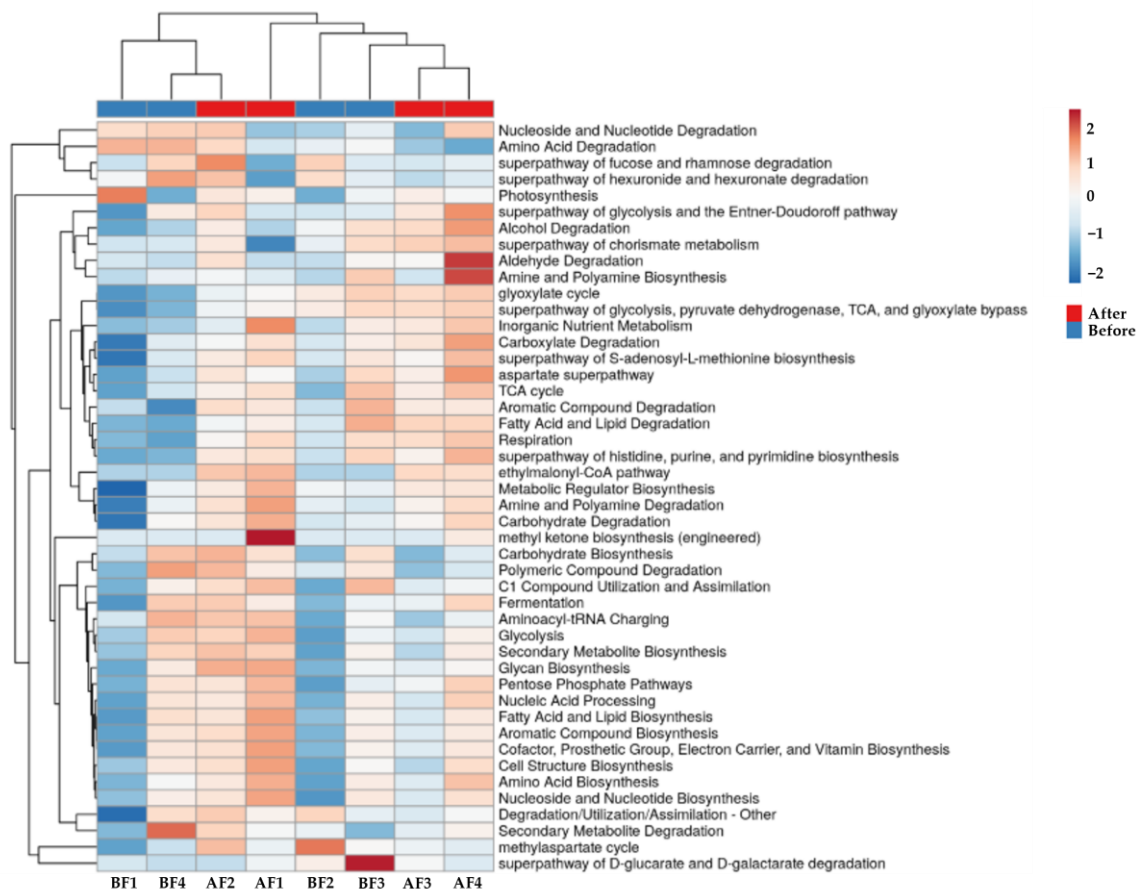

Supplement: Supplementary file 1 [file microorganisms-14-01347-s001.zip › Figures for submission Diakoumopoulou et al revised communiction supplementary.pdf]
